# Five-year efficacy and safety of repeated low-level red light therapy for myopia control in children: a multicenter, real-world study

**DOI:** 10.1016/j.aopr.2026.06.002

**Published:** 2026-06-12

**Authors:** Juzhao Zhang, Jun Yu, Haidong Zou, Yan Xu

**Affiliations:** a Shanghai Eye Diseases Prevention &Treatment Center/ Shanghai Eye Hospital, School of Medicine, Tongji University, Shanghai, China; b Shanghai Engineering Research Center of Precise Diagnosis and Treatment of Eye Diseases, Shanghai, China; c Eye Public Health Research Center, School of Medicine, Tongji University, Shanghai, China; d Xinhua Hospital, School of Medicine, Shanghai Jiao Tong University, Shanghai, China; e National Clinical Research Center for Eye Diseases, Shanghai General Hospital, School of Medicine, Shanghai Jiao Tong University, Shanghai, China

**Keywords:** Myopia, Repeated low-level red light, Children and adolescent, Control

## Abstract

**Objective:**

Repeated low-level red light (RLRL) therapy has emerged as a promising intervention for pediatric myopia control; however, large-scale evidence on its long-term efficacy and safety remains limited. This study aimed to evaluate the sustained effectiveness and safety of RLRL therapy in children and adolescents with myopia.

**Methods:**

This multicenter retrospective cohort study included patients aged 3–17 years who received RLRL therapy for at least 1 year across 12 clinical sites, with follow-up extending up to 5 years. The primary efficacy outcome was effective axial length (AL) control, defined as annual AL elongation <0.1 mm, assessed from years 1–5. Safety outcomes included the proportions of patients with a ≥2-line reduction in best-corrected visual acuity (BCVA) or BCVA worse than 20/25.

**Results:**

A total of 2885 patients were included. Effective AL control was observed in 53.8%–62.3% of patients across follow-up years, with consistent findings in sensitivity analyses. Efficacy peaked in year 1 and remained stable through year 5, with ≤5% annual fluctuation. Mean annual AL changes were 0.04, 0.12, 0.10, 0.11, and 0.08 mm in years 1 to 5, respectively. Control rates varied significantly by age, highest among patients aged 13–17 years and lowest among those aged 3–5 years (*P* < 0.05 for years 1–3). Baseline AL was associated with treatment outcomes, with 1-year control rates of 90.9% in the 28–31 mm group and 29.0% in the 21–23 mm group. Higher adherence was associated with better control (54.9%–63.5%) rates than lower adherence (25.0%–56.0%). No serious safety concerns were reported.

**Conclusions:**

In this large real-world cohort with follow-up of up to 5 years, RLRL therapy was associated with sustained AL control and no serious safety signals based on available clinical records. These findings support its potential role as a long-term myopia management strategy.

Myopia is one of the most prevalent visual disorders worldwide. According to the World Health Organization, the global prevalence of myopia has surpassed 2 billion, with a tendency toward earlier onset and progression to high myopia.^1^ The increasing prevalence of myopia, along with the irreversible visual impairment caused by myopia-related complications, presents a substantial burden on both individuals and society.^2^ Various intervention strategies have been developed for the prevention and control of myopia, including behavioral interventions, pharmaceutical treatments, and optical correction.^3^ However, most interventions exhibit significant inter-individual heterogeneity in efficacy,^4^ and the axial length (AL) control efficacy (currently recognized as the core indicator for myopia control) tends to diminish over time. In most cases, the efficacy decreases notably after 1–2 years of use, and in some instances, additional technologies must be combined to enhance myopia control.^5^, ^6^, ^7^, ^8^ Given that myopia progresses continuously during childhood and adolescence, its prevention and management require long-term, sustained treatment. Consequently, there is an urgent need to explore more effective intervention methods to address this growing public health challenge.

Repeated low-level red light (RLRL) therapy, an emerging method for myopia control, is gaining attention in the field of myopia control for children and adolescents. It exerts its core regulatory effect by simulating outdoor light exposure through red light of a specific wavelength around 650 nm,^9^^,^^10^ offering unique advantages such as non-invasiveness and ease of operation. A multicenter randomized controlled trial published in 2022 provided high-level evidence for the efficacy of RLRL in myopia control among school-aged children for the first time.^11^ Simultaneously, several clinical studies confirmed that RLRL effectively slows myopia progression in children and adolescents^12^, ^13^, ^14^, has a preventive effect on pre-myopic children,^15^ demonstrates favorable control efficacy even in those with high myopia,^16^ and is associated with significant AL shortening.^17^ Systematic reviews and meta-analyses further support RLRL's efficacy in controlling myopia progression, suggesting that its effect may surpass that of atropine and contact lenses.^18^ However, the follow-up periods of most studies are limited within 1–2 years, and some have noted that the control effect of RLRL in the second year of treatment is slightly weaker than in the first year.^19^ Whether the AL control efficacy will diminish over time remains unconfirmed due to a lack of long-term, large-sample follow-up data.

The safety of RLRL therapy, particularly as an intervention for healthy children and adolescents, has always been a primary concern. Existing research provides some reassurance regarding its safety. A systematic review and meta-analysis published in 2024 found no clear evidence that RLRL causes irreversible visual function impairment or ocular structural damage.^20^ However, one case study reported retinal damage in a Chinese myopic child following RLRL treatment.^21^ While this child may have had underlying congenital conditions, this case sparked significant discussions about the safety of RLRL.^22^ Recently, a research team analyzed the retinal structure of children who had received red light therapy for at least one year and identified one case with minor cystoid changes, which fully resolved three months after discontinuing treatment. Currently, clinical applications of RLRL emphasize the importance of combining fundus examinations, visual acuity monitoring, and afterimage duration recording to detect potential side effects early. However, safety evidence regarding visual acuity, lens, retina, intraocular pressure, and other indicators in long-term, large-sample follow-up studies of RLRL remain limited.

The purpose of this long-term real-world observational study is to retrospectively analyze the clinical data of patients receiving RLRL therapy across multiple centers, assess the efficacy and safety of its long-term application from 1 to 5 years, and provide additional evidence for the clinical application of RLRL as an emerging myopia control method.

## 1 Methods

### 1.1 Study design

This multicenter retrospective cohort study included myopic patients who received RLRL treatment for more than 1 year at 12 ophthalmic clinics across seven provinces (Shanghai, Jiangsu, Anhui, Guangdong, Zhejiang, Henan, and Inner Mongolia) from January 2018 to December 2024 (Supplementary Table 1). Participants received no financial compensation or other incentives.

For RLRL treatment, a desktop phototherapy device (Eyerising (Suzhou Xuanjia Photoelectric Technology Co., Ltd.)) was used. This device has been applied in amblyopia treatment in China for over a decade^23^ and has obtained Class IIa medical device certification from the National Medical Products Administration (NMPA) of China. It is also certified in several other countries/regions, including Europe (CE Mark Class IIa), the United Kingdom (UK MHRA Class IIa), New Zealand (MedSafe Class IIa), Turkey (TMMDA Class IIa), Australia (ARTG Class IIa), Malaysia (MDA Class B), and Vietnam (Vietnamese Ministry of Health Class B).

### 1.2 Inclusion and exclusion criteria

As a real-world observational study on RLRL treatment, this study used data from the earliest cases receiving red light therapy since 2017, with available follow-up records. To ensure statistical consistency, children and adolescents who met the following criteria were included: continuous RLRL treatment for ≥1 year from January 2018 to December 2024, aged 3–18 years, with a spherical equivalent (SE) < 0 D, and baseline examinations including best-corrected visual acuity (BCVA) and AL.

This study excluded only patients with incomplete follow-up data, with no additional exclusion criteria (including a history of ocular diseases) applied.

### 1.3 Data collection and adherence monitoring

Medical records from all participating centers were retrospectively reviewed, and data were systematically entered into electronic case report forms (eCRFs). Variables included demographic information, treatment initiation time, and during the follow-up period: uncorrected visual acuity (UCVA), BCVA, AL, SE, average corneal curvature (AVEK), central corneal thickness (CCT), anterior chamber depth (ACD), lens thickness (LT), intraocular pressure (IOP), combined myopia intervention measures (including single-vision spectacles, defocus incorporated multiple segments lenses (DIMS), and orthokeratology lenses (OK)), treatment adherence, and adverse events. The eCRFs incorporated a real-time verification function to minimize data entry errors. Assessments of the anterior segment and fundus were included in the recorded data.

To accurately assess adherence, treatment duration was automatically recorded by the device and synchronized with the server via the network. The system sent reminders to supervisors and participants who had missed treatment for two consecutive days. The server also monitored device voltage in real-time and remotely shut down the device in case of abnormal fluctuations. Additionally, for participants who discontinued treatment after more than one-year, manual telephone surveys were conducted to record the reasons for discontinuation. A total of 199 valid responses were obtained. The reasons for discontinuation included cost issues (46.2%), safety concerns (30.7%), inability to persist (20.1%), and other factors (3.0%, or 6 participants, all of whom had normal BCVA before discontinuation).

### 1.4 Study outcome

The outcome measures of interest focus on the efficacy and safety of red light therapy in myopia control:

(1) Efficacy: The primary outcome measure of efficacy is the effective AL control rate among patients who received RLRL treatment for 1, 2, 3, 4, and 5 years or more. Effective AL control was defined as an average annual AL elongation of <0.1 mm, based on previous studies and expert consensus.^24^^,^^25^ This threshold was selected as a conservative benchmark for substantial axial stabilization. AL was measured using optical biometers routinely used at each participating center (IOL Master 500, Carl Zeiss; Aladdin, Topcon; Lenstar LS 900, HAAG-STREIT). From baseline onward, each center used the same type of biometer for follow-up measurements. For each examination, three readings meeting quality-control requirements were obtained, and the mean value was used for analysis.

The secondary outcome measures included the average changes in non-cycloplegic SE each treatment year.^11^ All refractive error measurements were performed under small pupil conditions using autorefractometers (KR-8800, Topcon; KR-8900, Topcon; ARK-510A, Nidek Co Ltd). SE was calculated as the spherical power plus half the cylindrical power.

(2) Safety: With reference to previous study protocols, the primary safety outcomes include the proportion of patients experiencing a ≥2-line decrease in BCVA compared to baseline during red light therapy, and the proportion of patients with BCVA lower than 20/25.^11^ Distance visual acuity was measured using a Snellen chart.

The secondary outcome measures included subjectively reported adverse reactions during each treatment year.^17^ At every follow-up visit, the attending physician documented any adverse reactions reported by participants or their parents and conducted a fundus examination under a slit lamp to evaluate retinal conditions.

Exploratory indicators were used to supplement the description of relevant biological characteristics, including AVEK, CCT, ACD, LT, and IOP. AVEK, CCT, ACD, and LT were measured using the aforementioned ophthalmic biometers, while IOP was assessed using non-contact tonometers (CT-800, Topcon; Canon TX-20, Canon Inc).

### 1.5 Statistical analysis

Treatment duration was calculated in calendar days, standardized to 365 days per year, and grouped into annual intervals: 1 year (365–729 days), 2 years (730–1094 days), 3 years (1095–1459 days), 4 years (1460–1824 days), and ≥5 years (≥1825 days).

Sensitivity analysis was performed to assess the impact of RLRL on AL growth by adjusting the primary outcome threshold from <0.1 mm/year to <0.2 mm/year, comparing results across different thresholds to evaluate the stability of conclusions.

Adherence was calculated as the ratio of actual to recommended annual usage frequency for red light therapy. Based on prior studies, the recommended regimen was 3 min per session, twice daily (with ≥4-h intervals), 5 days per week.

For ophthalmic examination indicators, the annual value was the average of multiple follow-up measurements, and annual changes and change rates were calculated.

Quantitative data are presented as mean ± standard deviation, while categorical data are shown as frequencies and percentages. Inter-group comparisons were made using ANOVA, *t*-test, or rank-sum test for continuous data; chi-square test or Fisher's exact test for categorical data; and rank-sum test for ordinal data. Logistic regression and mixed-effects models were used to identify factors influencing RLRL's long-term efficacy.

Machine learning models, including random forest, XGBoost, gradient boosting decision tree, and support vector machine, were developed to predict long-term efficacy. Model inputs included age, gender, UCVA, SE, axis, BCVA, AL, AVEK, CCT, IOP, ACD, LT, and treatment regimen type. The output was classification based on annual average AL growth^25^: good response (<0.1 mm/year), stable response (0.1–0.2 mm/year), and poor response (>0.2 mm/year). Patients with ≥5 years of treatment was excluded due to small sample size. Parameter optimization was performed using RandomizedSearchCV, and 5-fold cross-validation validated the models. The area under the curve (AUC) and accuracy metrics were used to evaluate and select the optimal model. Statistical analyses were performed using SAS 9.4 and Python 3.11.8.

## 2 Results

### 2.1 Data distribution

The total sample size was 2885, with baseline distribution across study sites detailed in Supplementary Table 1. Among the duration-stratified groups, the 1-year group had the largest size (1037), followed by the 2-year (893), 3-year (568), 4-year (226), and ≥5-year (132) groups. Site 1 (1143), Site 11 (603), and Site 9 (432) were the major contributors, accounting for 75.5% of the total. Sites 2, 3, 4, 8, and 12 each had fewer than 100 participants.

### 2.2 Baseline characteristic

The study included 2674 right eyes from 2674 patients, with females representing 45.5% and a mean age of 9.42 ± 2.31 years at the start of red light therapy. Of the 2674 eyes, 2055 (76.9%) received RLRL alone, 536 (20.0%) received RLRL combined with DIMS, and 83 (3.1%) received RLRL combined with OK (Table 1).

**Table 1:** Baseline characteristics of participants involved in this study.

Total amount	n
Age, yrs	n = 2674
Mean ± SD	9.42 ± 2.31
Range	3.08–17.39
Age Group%	
3–5	6.3%
6–12	87.0%
13–17	6.7%
Gender	n = 2674
Female%	45.5%
Compliance	n = 2674
Compliance rate	0.80 ± 0.17
Compliance Group%	
75%–100%	1876 (70.2%)
50%–75%	575 (21.5%)
25%–50%	148 (5.5%)
0%–25%	75 (2.8%)
Combined treatment	n = 2674
RLRL	2055 (76.9%)
RLRL + DIMS	536 (20.0%)
RLRL + OK	83 (3.1%)
AL (mm), Mean ± SD	24.76 ± 1.16 (n = 2665)
AVEK, Mean ± SD	43.22 ± 1.52 (n = 2371)
Baseline Refraction	n = 2674
SER, D	−2.78 ± 2.49
Baseline SER Group	
2.0 D to 0.0 D	1174 (43.9%)
4.0 D to −2.0 D	773 (28.9%)
6.0 D to −4.0 D	435 (16.3%)
8.0 D to −6.0 D	292 (10.9%)
Baseline UCVA (logMAR), Mean ± SD	0.52 ± 0.30 (n = 970)
Baseline BCVA (logMAR), Mean ± SD	0.04 ± 0.12 (n = 2122)
Baseline IOP (mmHg), Mean ± SD	17.22 ± 2.65 (n = 1208)
Baseline CCT (μm), Mean ± SD	549.69 ± 38.24 (n = 90)
Baseline ACD (mm), Mean ± SD	3.30 ± 0.30 (n = 46)
Baseline LT (mm), Mean ± SD	3.48 ± 0.24 (n = 62)

### 2.3 Efficacy of RLRL therapy in myopia control

As shown in Fig. 1 and Table 2, RLRL demonstrated long-term efficacy in controlling myopia progression, with the effective control rate of AL growth (<0.1 mm/year) remaining between 53.8% and 62.3% across years.

**Fig. 1:**
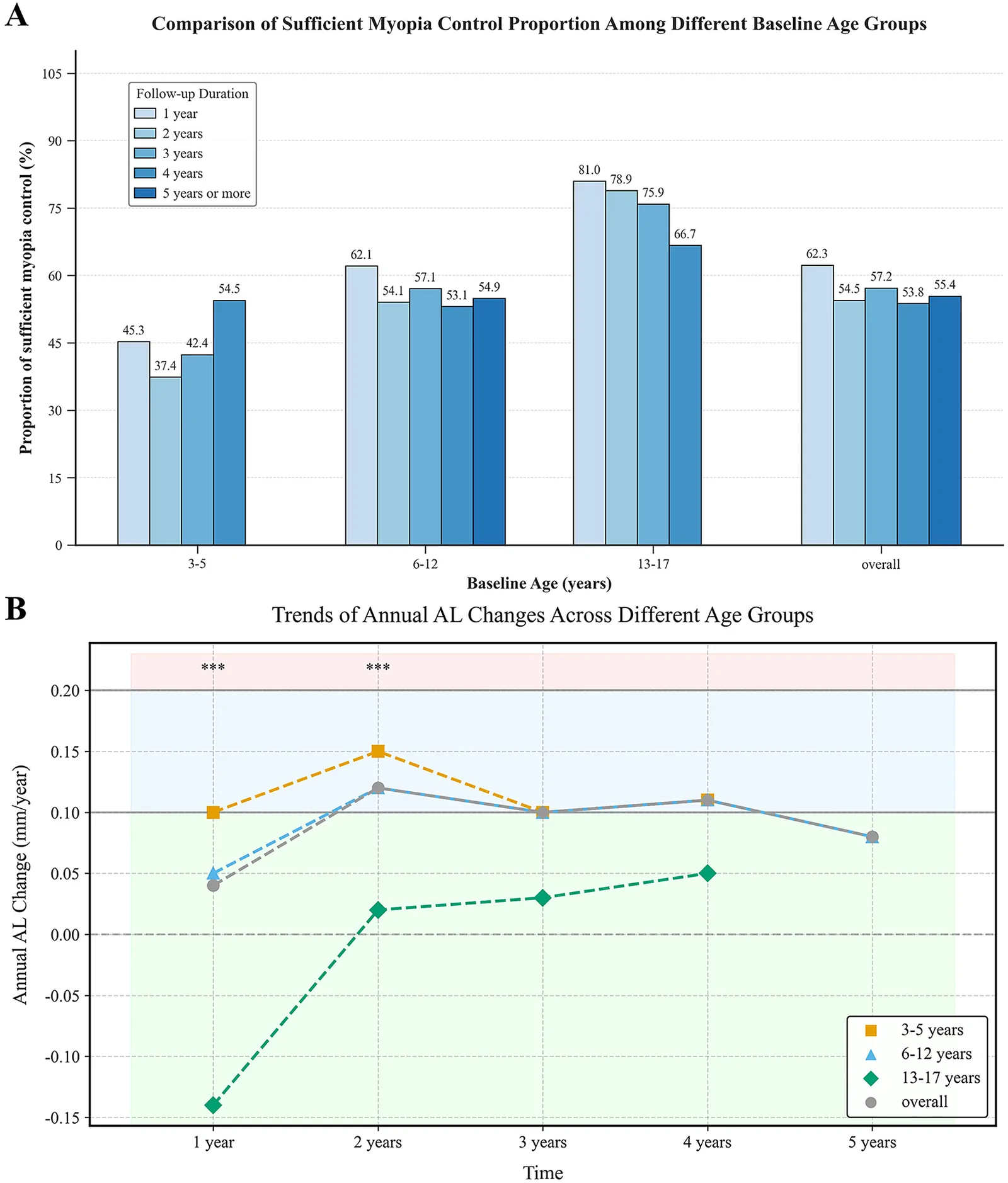
Myopia control efficacy and annual axial length change across baseline age groups by follow-up duration. (A) Myopia control efficacy rates across different baseline age groups by follow-up duration. The x-axis represents baseline age groups, the y-axis indicates efficacy rates, and data are stratified by follow-up duration. Efficacy was defined as effective control (annual average AL growth <0.1 mm). (B) Annual ΔAL trends across age groups. It presents ΔAL trends of different baseline age groups over follow-up durations; background colors indicate ΔAL ranges: green (<0.1 mm/year), blue (0.1–0.2 mm/year), red (>0.2 mm/year). Two subgroups were omitted: 3–5 years and 13–17 years age groups (both with ≥5 years of follow-up, n = 1 and n = 2, respectively), due to risk of chance overestimation given the data do not represent the true population effect.

**Table 2:** The proportion of axial length growth that is effectively controlled (<0.1 mm/year).

AL control ratio from baseline	1 year (n, %)	2 years (n, %)	3 years (n, %)	4 years (n, %)	5 years or more (n, %)
**Overall**	1616, 62.3%	826, 54.5%	362, 57.2%	106, 53.8%	31, 55.4%
**Baseline Age**					
3–5	73, 45.3%	37, 37.4%	14, 42.4%	6, 54.5%	2, 50.0%
6–12	1398, 62.1%	718, 54.1%	326, 57.1%	94, 53.1%	28, 54.9%
13–17	145, 81.0%	71, 78.9%	22, 75.9%	6, 66.7%	1, 100%
*P*	<0.001	<0.001	<0.05	0.7782	0.8347
**Gender Group**					
Male	881, 62.7%	441, 53.1%	185, 54.4%	56, 51.4%	21, 58.3%
Female	735, 61.8%	385, 56.3%	177, 60.6%	50, 56.8%	10, 50.0%
*P*	0.2293	0.4540	0.2461	0.6264	0.8260
**AL Group**					
21–23	31, 29.0%	20, 29.9%	9, 56.3%	2, 66.7%	2, 100.0%
23–25	832, 55.7%	412, 48.2%	195, 53.3%	60, 52.6%	17, 51.5%
25–28	733, 75.6%	385, 66.0%	155, 62.8%	44, 55.0%	12, 57.1%
28–31	20, 90.9%	9, 81.8%	3, 75.0%	/	/
*P*	<0.001	<0.001	0.0774	0.9635	0.9423
**Compliance Group**					
≥50%	1532, 63.5%	789, 54.9%	348, 57.2%	104, 55.0%	31, 57.4%
<50%	84, 46.2%	37, 48.1%	14, 56.0%	2, 25.0%	/
*P*	<0.001	0.0565	0.8643	0.2367	<0.05
**Treatment Group**					
RLRL	1244, 62.6%	618, 53.7%	273, 55.5%	88, 52.7%	23, 51.1%
RLRL + DIMS	326, 62.1%	176, 57.1%	77, 65.3%	11, 50.0%	6, 66.7%
RLRL + OK	46, 56.8%	32, 57.1%	12, 52.2%	7, 87.5%	2, 100.0%
*P*	<0.05	<0.05	0.1469	0.7293	0.5241

For patients with ≤3 years of treatment, the 13–17-year baseline age group had the highest control rate (66.7%–100%), the 3–5-year group the lowest (37.4%–54.5%), and the 6–12-year group fell in between. Control rates were comparable between males (51.4%–62.7%) and females (50.0%–61.8%).

Across intervention strategies, the RLRL and RLRL + DIMS groups had relatively higher overall control rates (62.6%; 62.1%), with no significant differences between groups during long-term use. Sensitivity analysis using alternative outcome definition criteria confirmed the robustness of the main results (Supplementary Table 2).

In the first 3 years of RLRL use, SE slightly regressed in Year 1 (0.13 ± 1.53), followed by progression in Years 2–3 (−0.17 ± 1.20; −0.15 ± 1.12), with significant differences in annual average change between adjacent years. For ≥4 years of use, no statistical differences were observed (−0.21 ± 1.57; −0.12 ± 1.22). AVEK followed a similar pattern, with significant differences in the first 3 years (−0.07 ± 0.51; −0.09 ± 0.37; −0.07 ± 0.42) but not thereafter (−0.00 ± 0.51; −0.07 ± 0.37). Mean values remained relatively stable across time points.

Lost-to-follow-up analysis (Supplementary Table 3) showed no significant differences in baseline characteristics, including age, gender, and AL, between participants with complete follow-up and those with missing data from years 2 to 5 (*P* > 0.01), suggesting limited baseline imbalance related to follow-up status.

### 2.4 Predictors of long-term efficacy of RLRL therapy

Multivariate logistic regression showed that after 1 year of treatment, older baseline age, poorer adherence, and longer baseline AL were associated with higher risk of annual AL growth >0.2 mm. With longer treatment, baseline age remained the only consistent predictor, suggesting a broad applicability of RLRL (Supplementary Table 4). In patients with varying degrees of AL shortening, predictors included age, adherence, baseline AL, and baseline SE (Supplementary Table 5, 6).

Mixed-effects modeling indicated that annual AL change was influenced by treatment year, age, adherence, baseline AL, and baseline SE. The best control effect occurred after 1 year, with mean AL growth of ∼0.05 mm; in subsequent years, annual growth averaged ∼0.1 mm, still within effective control. Better adherence was consistently associated with slower AL progression (Supplementary Table 7).

Machine learning models using biometric and demographic data achieved AUC values of 0.565–0.689 for predicting long-term efficacy, indicating modest discriminative ability (Supplementary Table 8, Supplementary Fig.1, 2).

### 2.5 Safety of RLRL therapy in myopia control

During long-term treatment, visual acuity was generally maintained, and no definite evidence of treatment-related fundus structural damage or adverse reactions was identified in the available clinical records (Table 3, Supplementary Table 9).

**Table 3:** Changes in BCVA for RLRL treatment.

	1 year, (n , %)	2 years, (n , %)	3 years, (n , %)	4 years, (n, %)	5 years or more, (n, %)
**Change in BCVA**					
2 lines worse	4, 0.2%	3, 0.2%	1, 0.2%	0	0
within 1 line	1625, 88.2%	1183, 98.3%	512, 99.2%	146, 98.0%	37, 94.9%
2 lines better	214, 11.6%	18, 1.5%	3, 0.6%	3, 2.0%	2, 5.1%
**BCVA**					
<0.8	101, 4.8%	50, 3.9%	17, 3.1%	2, 1.3%	1, 2.4%
Proportion of baseline <0.8	69, 68.4%	35, 70%	8, 47.1%	1, 50%	1, 100%

Most patients had stable BCVA or a ≤1-line decrease. Only 8 cases showed a ≥2-line decrease: 4 occurred in Year 1, of which 2 recovered with continued treatment and 2 withdrew; 3 occurred in Year 2, all of whom withdrew; and 1 occurred in Year 3 in a patient with high myopia patient and under-corrected vision at baseline (OD: −14.00 DS/−4.5 DC; OS: −13.50 DS/−4.00 DC; AL: 29.44/29.47 mm). This case maintained a minimum BCVA of 0.7, and the decrease was not considered clearly attributable to RLRL treatment based on the available records.

The proportion of patients with BCVA <0.8 remained low (1.3%–4.8%). Most of these patients (68%) already had BCVA <0.8 at baseline and showed no further decline. Of the remainder, apart from the aforementioned ≥2-line cases, the rest had baseline BCVA of 0.9 and decreased by only 1 line, which may reflect measurement variability or transient visual fluctuation rather than a definite treatment-related safety signal.

Among patients with BCVA <0.8 at Year 4 or ≥ Year 5, only two cases were identified. One was a 6-year-old who entered the study with poor baseline vision (UCVA 0.2/0.2; BCVA 0.3/0.3) and high myopia (OD: −10.25 DS/−3.75 DC; OS: −10.50 DS/−4.50 DC; AL: 26.10/26.34 mm), suggestive of congenital high myopia with amblyopia. The other was a 9-year-old with good baseline vision (BCVA 0.8/0.8) who maintained stable acuity for 3 years; BCVA declined to 0.6/0.8 in Year 4 but recovered spontaneously in Year 5, suggesting a possible functional abnormality.

## 3 Discussion

This study represents a large multicenter real-world retrospective analysis with long-term follow-up of RLRL therapy for myopia control. Results showed 53.8%–62.3% of patients achieved effective AL control, defined as annual AL growth <0.1 mm, and the findings remained consistent in sensitivity analysis when the threshold was adjusted to <0.2 mm. RLRL efficacy was greatest in the first year and subsequently appeared to enter a plateau phase from the second year onward, with the control rate declining from 62.3% to 55.4%. This pattern is consistent with a previous retrospective study,^19^ which reported mean AL growth of 0.04 mm in the first year and 0.12 mm in the second year. The longer follow-up in the present cohort further suggests that this plateau pattern may persist through year 5, with annual fluctuations of ≤5% and no significant decline among patients with available follow-up.

Annual AL changes in RLRL-treated patients were 0.04, 0.12, 0.10, 0.11, and 0.08 mm at 1, 2, 3, 4, and ≥5 years, respectively. Compared with atropine, RLRL may provide superior long-term control: a 36-month trial reported annual AL growth of 0.23 mm with 0.01% atropine and 0.24 mm with 0.02% atropine^26^; another study found cumulative AL increases of 0.05, 0.24, 0.47, and 0.56 mm over 1–4 years with 1% atropine, with ∼0.20 mm annual growth from Year 2 onward.^5^ However, further studies are needed to confirm this advantage.

Age-stratified data showed the highest control rate in the 13–17 years group, followed by the 6–12 years group, and the lowest in the 3–5 years group, with significant differences in the first three years (*P* < 0.05). These outcomes reflect physiological AL growth pattern: children aged 3–5 years undergo rapid natural AL elongation, while growth stabilizes in adolescents aged 13–17 years, contributing to stronger treatment responses. Notably, the myopia control rate in the 6–12 years group still showed a downward trend over time, suggesting no interference from survival bias.

Analysis by baseline AL revealed that longer AL correlated with higher control rates: the 1-year control rate reached 90.9% in the 28–31 mm group, versus only 29.0% in the 21–23 mm group (*P* < 0.001). This is consistent with previous findings that RLRL is more effective in high myopia.^16^ A possible mechanism is that high myopia is associated with thinner choroids and reduced retinal microvascular density; RLRL may induce greater increases in choroidal and retinal thickness in these patients, thus contributing to more significant AL control effects.

Adherence analysis showed higher annual control rates in the high-adherence group (54.9%–63.5%) than in the low-adherence group (25.0%–56.0%), consistent with earlier reports.^11^ Notably, adherence as low as 50% still provided favorable effects, suggesting lower treatment frequency may suffice. Prior studies also found that RLRL at doses of 0.37, 0.60, and 1.20 mW all slowed myopia progression, with no significant differences among groups.^14^ These results collectively suggest that the optimal frequency and dose of RLRL for myopia control warrant further exploration.

Given the real-world nature of this study, concurrent use of other myopia-control interventions was observed in a subset of participants, providing an opportunity to describe treatment patterns and outcomes in routine clinical practice. Short-term control rates varied across treatment subgroups, broadly consistent with previous clinical observations^27^, ^28^, ^29^, whereas long-term effects tended to converge. Low-concentration atropine was not combined with RLRL in this cohort because atropine-induced pupillary dilation may increase fundus light exposure and is not routinely recommended in clinical practice. Nevertheless, these findings should be interpreted as non-comparative observations rather than direct evidence of superiority among interventions. In addition, the real-world setting should also be considered when interpreting refractive outcomes. Although cycloplegic refraction is generally preferred in pediatric myopia studies to minimize accommodative influence, repeated cycloplegic examinations are difficult to implement consistently in long-term multicenter routine practice and may reduce acceptance and adherence among children and parents. Therefore, non-cycloplegic SE may produce a more myopic estimate, particularly in younger children with stronger accommodative responses, and SE-related findings should be interpreted cautiously. Importantly, as the primary efficacy endpoint was AL control rather than SE progression, the use of non-cycloplegic SE is unlikely to affect the main conclusion.

Machine learning models yielded AUC values of 0.565–0.689 for predicting long-term efficacy (Supplementary Table 8), lower than the 0.710–0.786 reported by Xiong et al.,^25^ likely because their study focused on 1-year efficacy and included 3-month macular thickness data unavailable here. Xiong et al. also found limited additional benefit from other biometric and demographic features, suggesting retinal structural changes may have unique predictive value. Results of feature importance analysis showed (Supplementary Fig.1, 2) that baseline age, baseline AL, and adherence consistently ranked among the top key features, aligning with stratified results.

Adverse reactions reported in previous studies were generally mild and transient, including blurred vision, dry eyes, soreness, or visual fatigue, while eye pain or mild stinging was less common and rarely led to discontinuation. In the present study, no definite treatment-related adverse reactions, sustained visual loss, or fundus damage were identified from the available follow-up records, and parameters such as IOP, CCT, ACD, LT, and AVEK remained generally stable. Nevertheless, 30.7% of discontinuations were due to safety concerns, underscoring the need for strict adherence to standardized protocols. Future prospective studies with standardized safety assessments are needed to more rigorously evaluate the long-term safety profile of RLRL therapy.

This study has several limitations. First, real-world retrospective observational design is inherently prone to bias. Participants were clinical RLRL patients with completed follow-up, and the absence of parallel controls limits adjustment for potential confounders such as natural myopia progression. In addition, important environmental and behavioral variables, including outdoor exposure, near-work habits, screen time, and educational intensity, were not available in this retrospective dataset. Second, the follow-up sample size declined over time, particularly in later years. Although lost-to-follow-up analysis showed no major baseline differences, the reduced sample size may limit the reliability of long-term estimates. Third, cycloplegic refraction was not performed, so BCVA may have been influenced by accommodation. Finally, the study population was limited to Chinese children and adolescents aged 3–18 years, restricting generalizability to other ethnic groups.

## 4 Conclusions

In this long-term real-world observational study, we for the first time present 5-year follow-up data of nearly 3000 children and adolescents, demonstrating the stable efficacy and safety of RLRL in long-term myopia control. This provides an evidence base for the clinical promotion of this emerging therapy.

## Study approval

The authors confirm that any aspect of the work covered in this manuscript that involved human patients or animals was conducted with the ethical approval of all relevant bodies and the study was performed in accordance with the Declaration of Helsinki，and the protocol was approved by the Ethics Committee of Shanghai Eye Disease Prevention and Treatment Center (EC-20240913-01).

## Author contributions

The authors confirm contribution to the paper as follows: Conceptualization: JZ, HZ and YX; Formal analysis, Writing – original draft: JZ, JY and YX; Writing – review and editing: HZ, YX. All authors approved the final manuscript.

## Funding

This work was supported by grants from the Special Program for Clinical Research in Healthcare of 10.13039/100017950Shanghai Municipal Health Commission (No. 202540067), the Clinical Technology Promotion & Optimization Management Project of 10.13039/501100014137Shanghai Shenkang Hospital Development Center (No. SHDC12025102), the Shanghai Science and Technology Innovation Action Plan Young Scientist Star Program (No. 24YF2740300) and the Postdoctoral Fellowship Program (No. GZB20250556).

## Declaration of competing interest

The authors declare that they have no known competing financial interests or personal relationships that could have appeared to influence the work reported in this paper.
