# Supplementary material for: Five-year efficacy and safety of repeated low-level red light therapy for myopia control in children: a multicenter, real-world study
Source: Adv Ophthalmol Pract Res. 2026 Jun 12;6(4):389–94. doi: 10.1016/j.aopr.2026.06.002 (PMC13770577; doi:10.1016/j.aopr.2026.06.002)
Supplement: Supplementary file 1 [file mmc1.docx]

**Supplementary Information**

**Supplementary Table 1. Participant distribution at baseline across 12 study sites**

**Supplementary Table 2. Sensitivity analysis (Proportion of axial length growth <0.2 mm/year)**

**Supplementary Table 3. Comparison of baseline characteristics between dropout groups and retained groups in each year**

**Supplementary Table 4. Logistic model for poor response groups after 1 year, 2 years, and 3 years of continuous use**

**Supplementary Table 5. Logistic model for axial length shortening after 1 year of continuous use**

**Supplementary Table 6. Logistic model for axial length shortening after 2 years of continuous use**

**Supplementary Table 7. Factors affecting RLRL-mediated control of axial length growth**

**Supplementary Table 8. Performance of machine learning models for long-term efficacy prediction across years 1-4**

**Supplementary Table 9. Changes in exploratory outcomes during the treatment period.**

**Supplementary Fig.1. Feature importance plots of the optimal model for Years 1–4**

**Supplementary Fig.2. Alluvial diagrams for model prediction results and processes.**

Supplementary Table 1. Participant distribution at baseline across 12 study sites

| Site | < 1 year | 1 year | 2 years | 3 years | 4 years | 5 years or more | Total |
| --- | --- | --- | --- | --- | --- | --- | --- |
| Site 1 | 12 | 377 | 396 | 214 | 86 | 58 | 1143 |
| Site 2 |  |  |  | 9 | 10 | 12 | 31 |
| Site 3 |  | 3 | 5 | 5 | 7 | 9 | 29 |
| Site 4 |  |  | 1 | 3 | 3 | 3 | 10 |
| Site 5 |  |  |  |  |  | 15 | 15 |
| Site 6 | 4 | 26 | 44 | 71 | 88 | 29 | 262 |
| Site 7 | 1 | 132 | 88 | 39 |  |  | 260 |
| Site 8 |  | 1 | 1 | 2 |  |  | 4 |
| Site 9 | 1 | 179 | 143 | 109 |  |  | 432 |
| Site 10 |  | 7 | 11 | 15 | 7 | 3 | 43 |
| Site 11 | 11 | 307 | 173 | 84 | 25 | 3 | 603 |
| Site 12 |  | 5 | 31 | 17 |  |  | 53 |
| Total | 29 | 1037 | 893 | 568 | 226 | 132 | 2885 |

*Note: Site 1: Aierxing Service Center, Anhui Province; Site 2: Hangzhou Huiai Vision Clinic, Zhejiang Province; Site 3: Anyang Tongguang Pediatric Ophthalmology, Henan Province; Site 4: Anyang Tongguang Optometry Center, Henan Province; Site 5: The First Affiliated Hospital of Baotou Medical College, Inner Mongolia University of Science and Technology; Site 6: Shanghai Heping Ophthalmic Hospital; Site 7: Shanghai Aierxing Service Center; Site 8: Shanghai Mingtan Ophthalmic Hospital; Site 9: Shenzhen Eye Hospital; Site 10: Suliping Aierxing Service Center; Site 11: Lixiang eye hospital of soochow university; Site 12: Suzhou Aierxing Service Center*

Supplementary Table 2. Sensitivity analysis (Proportion of axial length growth <0.2 mm/year)

| AL growth <0.2mm/y | **1 year (N, %)** | **2 years (N, %)** | **3 years (N, %)** | **4 years (N, %)** | **5 years or more (N,%)** |
| --- | --- | --- | --- | --- | --- |
| **Overall** | **1967, 75.9%** | **1205, 79.5%** | **530, 83.7%** | **162, 82.2%** | **44, 78.6%** |
| **Baseline Age** |  |  |  |  |  |
| 3–5 | 95, 59.0% | 76, 76.8% | 27, 81.8% | 10, 90.9% | 3, 75.0% |
| 6–12 | 1709, 75.9% | 1039, 78.4% | 476,83.4% | 144, 81.4% | 40, 78.4% |
| 13–17 | 163, 91.1% | 90, 100.0% | 27, 93.1% | 8, 88.9% | 1, 100% |
| **Gender Group** |  |  |  |  |  |
| Male | 1061, 75.6% | 657, 79.1% | 282, 82.9% | 90, 82.6% | 29, 80.6% |
| Female | 906, 76.2% | 548, 80.1% | 248, 84.9% | 72, 81.8% | 15, 75.0% |
| **Treatment Group** |  |  |  |  |  |
| RLRL | 1512, 76.1% | 908, 78.9% | 409, 83.1% | 139, 83.2% | 34, 75.6% |
| RLRL+DIMS | 397, 75.6% | 251, 81.5% | 102, 86.4% | 16, 72.7% | 8, 88.9% |
| RLRL+OK | 58, 71.6% | 46, 82.1% | 19, 82.6% | 7, 87.5% | 2, 100.0% |
| **Compliance Group** |  |  |  |  |  |
| >=50% | 1856, 77.0% | 1152, 80.1% | 510, 83.9% | 156, 82.5% | 44, 81.5% |
| <50% | 111, 61.0% | 53, 68.8% | 20, 80.0% | 6, 75.0% | / |
| **AL Group** |  |  |  |  |  |
| 21–23 | 49, 45.8% | 43, 64.2% | 13, 81.3% | 3, 100.0% | 2, 100.0% |
| 23–25 | 1063, 71.2% | 648, 75.9% | 302, 82.5% | 93, 81.6% | 25, 75.8% |
| 25–28 | 835, 86.1% | 504, 86.4% | 212, 85.8% | 66, 82.5% | 17, 81.0% |
| 28–31 | 20, 90.9% | 10, 90.9% | 3, 75.0% | / | / |

Supplementary Table 3. Comparison of baseline characteristics between dropout groups and retained groups in each year

| **Characteristic** | **Dropout patients** | **Retained patients** | **p** |
| --- | --- | --- | --- |
| 2 years | N = 134 | N = 1459 |  |
| **Age, yrs** |  |  |  |
| Mean ± SD | 9.06±2.00 | 9.35±2.26 | 0.1514 |
| **Gender, no. (%)** |  |  |  |
| Male | 71 (53.0) | 797 (54.7) | 0.7056 |
| Female | 63 (47.0) | 662 (45.3) |  |
| **AL, mm** |  |  |  |
| Mean ± SD | 24.71±1.01 | 24.79±1.15 | 0.4100 |
| **AL group, no. (%)** |  |  |  |
| 21-23 | 2 (1.5) | 54 (3.7) | 0.3772 |
| 23-25 | 86 (64.2) | 845 (57.9) |  |
| 25-28 | 45 (33.6) | 545 (37.4) |  |
| 28-31 | 1 (0.7) | 15 (1.0) |  |
| **Age group, no. (%)** |  |  |  |
| 3-5 | 3 (2.2) | 88 (6.0) | 0.1580 |
| 6-12 | 124 (92.5) | 1278 (87.6) |  |
| 13-17 | 7 (5.2) | 93 (6.4) |  |
|  |  |  |  |
| 3 years | N = 144 | N = 625 |  |
| **Age, yrs** |  |  |  |
| Mean±SD | 9.65± 2.43 | 9.49± 2.26 | 0.4546 |
| **Gender, no. (%)** |  |  |  |
| Male | 70 (48.6) | 342 (54.9) | 0.1728 |
| Female | 74 (51.4) | 283 (45.1) |  |
| **AL, mm** |  |  |  |
| Mean ± SD | 24.89±1.09 | 24.88±1.17 | 0.9631 |
| **Age group, no. (%)** |  |  |  |
| 3-5 | 9 (6.3) | 32 (5.1) | 0.1829 |
| 6-12 | 119 (82.6) | 550 (88.0) |  |
| 13-17 | 16 (11.1) | 43 (6.9) |  |
| **AL group, no. (%)** |  |  |  |
| 21-23 | 1 (0.7) | 17 (2.7) | 0.3205 |
| 23-25 | 89 (61.8) | 347 (55.5) |  |
| 25-28 | 53 (36.8) | 254 (40.6) |  |
| 28-31 | 1 (0.7) | 7 (1.1) |  |
|  |  |  |  |
| 4 years | N = 135 | N = 303 |  |
| **Age, yrs** |  |  |  |
| Mean±SD | 9.93± 2.52 | 9.49± 2.26 | 0.0501 |
| **Gender, no. (%)** |  |  |  |
| Male | 67 (49.6) | 160 (52.8) | 0.5391 |
| Female | 68 (50.4) | 143 (47.2) |  |
| **AL, mm** |  |  |  |
| Mean ± SD | 25.06±1.28 | 24.79±1.15 | 0.0301 |
| **Age group, no. (%)** |  |  |  |
| 3-5 | 8 (5.9) | 14 (4.6) | 0.0133 |
| 6-12 | 106 (78.5) | 268 (88.4) |  |
| 13-17 | 21 (15.6) | 21 (6.9) |  |
| **AL group, no. (%)** |  |  |  |
| 21-23 | 2 (1.5) | 8 (2.6) | 0.5961 |
| 23-25 | 76 (56.3) | 179 (59.1) |  |
| 25-28 | 54 (40.0) | 113 (37.3) |  |
| 28-31 | 3 (2.2) | 3 (1.0) |  |
|  |  |  |  |
| 5 years | N = 40 | N = 81 |  |
| **Age, yrs** |  |  |  |
| Mean±SD | 9.79± 2.53 | 9.15± 2.24 | 0.1592 |
| **Gender, no. (%)** |  |  |  |
| Male | 17 (42.5) | 43 (53.1) | 0.2732 |
| Female | 23 (57.5) | 38 (46.9) |  |
| **AL, mm** |  |  |  |
| Mean ± SD | 24.87±1.06 | 24.69±0.99 | 0.3673 |
| **Age group, no. (%)** |  |  |  |
| 3-5 | 2 (5.0) | 4 (4.9) | 0.8502 |
| 6-12 | 35 (87.5) | 73 (90.1) |  |
| 13-17 | 3 (7.5) | 4 (4.9) |  |
| **AL group, no. (%)** |  |  |  |
| 21-23 | / | 1 (1.2) | 0.7511 |
| 23-25 | 27 (56.3) | 52 (64.2) |  |
| 25-28 | 13 (40.0) | 28 (34.6) |  |
| 28-31 | / | / |  |

Supplementary Table 4. Logistic model for poor response groups after 1 year, 2 years, and 3 years of continuous use

| **Factors** | **＞0.20mm/year** | | **＞ 0.20mm/year** | | **＞ 0.20mm/year** | |
| --- | --- | --- | --- | --- | --- | --- |
|  | **OR** | **P** | **OR** | **P** | **OR** | **P** |
| Gender Female-Male | 0.854(0.683,1.068) | 0.1675 | 0.835(0.630,1.107) | 0.2105 | 0.876(0.546,1.406) | 0.5845 |
| age | 1.216(1.148,1.287) | **<.0001** | 1.195(1.111,1.284) | **<.0001** | 1.151(1.022,1.298) | **0.0209** |
| compliance 4-1 | 0.737(0.381,1.425) | **0.0380** | 0.438(0.154,1.242) | 0.2245 | 0.000(0.000, I) | 0.9843 |
| compliance 4-2 | 0.462(0.234,0.913) | **0.0807** | 0.471(0.161,1.377) | 0.4987 | 0.000(0.000, I) | 0.9823 |
| compliance 4-3 | 0.334(0.153,0.727) | **0.0033** | 0.398(0.124,1.271) | 0.2324 | 0.000(0.000, I) | 0.9843 |
| al1_0 | 1.556(1.269,1.908) | **<.0001** | 1.085(0.843,1.395) | 0.5261 | 0.983(0.651,1.482) | 0.9337 |
| ser1_0 | 0.957(0.884,1.037) | 0.2836 | 0.931(0.843,1.027) | 0.1545 | 0.963(0.823,1.127) | 0.6369 |
| colens 1-2 | 1.099(0.825,1.464) | 0.4395 | 0.813(0.562,1.177) | 0.3301 | 0.498(0.237,1.045) | 0.3061 |
| colens 1-3 | 1.071(0.757,1.515) | 0.6699 | 1.012(0.641,1.597) | 0.5665 | 0.592(0.252,1.393) | 0.8553 |
| colens 1-4 | 0.908(0.501,1.646) | 0.5987 | 0.880(0.401,1.932) | 0.8684 | 0.506(0.131,1.948) | 0.6570 |
| avek1_0 | 1.080(0.977,1.193) | 0.1311 | 0.973(0.859,1.103) | 0.6728 | 0.978(0.793,1.205) | 0.8322 |

Supplementary Table 5.  Logistic model for axial length shortening after 1 year of continuous use

| **Factors** | **＞ -0.05mm/year** | | **＞-0.10mm/year** | | **＞ -0.20mm/year** | |
| --- | --- | --- | --- | --- | --- | --- |
|  | **OR** | **P** | **OR** | **P** | **OR** | **P** |
| Gender | 1.160(0.944,1.426) | 0.1579 | 1.149(0.922,1.433) | 0.2168 | 1.115(0.857,1.449) | 0.4178 |
| Age | 0.820(0.781,0.862) | **<.0001** | 0.813(0.772,0.855) | **<.0001** | 0.813(0.767,0.863) | **<.0001** |
| Compliance | 0.150(0.083,0.271) | **<.0001** | 0.145(0.077,0.275) | **<.0001** | 0.100(0.045,0.224) | **<.0001** |
| Baseline AL | 0.723(0.632,0.827) | **<.0001** | 0.737(0.640,0.848) | **<.0001** | 0.766(0.650,0.903) | **0.0015** |
| Baseline SE | 1.102(1.041,1.167) | **0.0008** | 1.128(1.062,1.198) | **<.0001** | 1.176(1.096,1.261) | **<.0001** |
| RLRL+DIMS | 0.768(0.554,1.063) | 0.7324 | 0.756(0.532,1.072) | 0.6724 | 0.767(0.500,1.176) | 0.4536 |
| RLRL+OK | 0.731(0.412,1.299) | 0.6722 | 0.713(0.384,1.321) | 0.6200 | 0.981(0.418,2.301) | 0.6656 |

Supplementary Table 6. Logistic model for axial length shortening after 2 years of continuous use

| **Factors** | **＞ -0.05mm/year** | | **＞-0.10mm/year** | | **＞ -0.20mm/year** | |
| --- | --- | --- | --- | --- | --- | --- |
|  | **OR** | **P** | **OR** | **P** | **OR** | **P** |
| Gender | 1.197(0.975,1.468) | 0.0853 | 1.165(0.948,1.433) | 0.1465 | 1.143(0.929,1.407) | 0.2057 |
| Age | 0.860(0.818,0.903) | <.0001 | 0.864(0.822,0.908) | <.0001 | 0.862(0.820,0.907) | <.0001 |
| Compliance | 1.723(0.961,3.088) | 0.0676 | 1.735(0.964,3.120) | 0.0659 | 1.730(0.961,3.115) | 0.0679 |
| Baseline AL | 0.955(0.839,1.087) | 0.4822 | 0.982(0.862,1.119) | 0.7878 | 0.999(0.876,1.140) | 0.9935 |
| Baseline SE | 1.057(0.998,1.119) | 0.0593 | 1.059(0.999,1.122) | 0.0544 | 1.059(0.999,1.123) | 0.0555 |
| RLRL+DIMS | 1.157(0.844,1.587) | 0.7196 | 1.182(0.860,1.623) | 0.6691 | 1.175(0.855,1.616) | 0.5915 |
| RLRL+OK | 1.332(0.742,2.394) | 0.6356 | 1.461(0.803,2.658) | 0.4524 | 1.516(0.826,2.780) | 0.3817 |

Supplementary Table 7. Factors affecting RLRL-mediated control of axial length growth

| **Independent variable** | **Estimation** | **Lower limit** | **Upper limit** | **p** |
| --- | --- | --- | --- | --- |
| Intercept | 1.4318 | 1.2043 | 1.6593 | <0.001 |
| Follow-up year 1-0 | 0.05730 | 0.05071 | 0.06388 | **<0.001** |
| Follow-up year 2-0 | 0.1474 | 0.1377 | 0.1571 | **<0.001** |
| Follow-up year 3-0 | 0.2447 | 0.2310 | 0.2583 | **<0.001** |
| Follow-up year 4-0 | 0.3449 | 0.3241 | 0.3657 | **<0.001** |
| Follow-up year 5-0 | 0.4560 | 0.4230 | 0.4889 | **<0.001** |
| Follow-up year 6-0 | 0.5932 | 0.5138 | 0.6725 | **<0.001** |
| Follow-up year 7-0 | 0.6238 | 0.3932 | 0.8544 | **<0.001** |
| Age | -0.01350 | -0.01719 | -0.00981 | **<0.001** |
| Sex Female-Male | 0.006074 | -0.01662 | 0.02876 | 0.4986394 |
| Compliance | -0.1779 | -0.2259 | -0.1299 | **<0.001** |
| Baseline AL | 0.9530 | 0.9431 | 0.9629 | **<0.001** |
| Baseline SER | 0.006873 | 0.002732 | 0.01101 | **<0.001** |

Supplementary Table 8. Performance of machine learning models for long-term efficacy prediction across years 1-4

| **Model** | **year1_AUC** | **year2_AUC** | **year3_AUC** | **year4_AUC** |
| --- | --- | --- | --- | --- |
| Random Forest | 0.6891±0.0070 | 0.6456 ± 0.0373 | 0.5689 ± 0.0242 | 0.5645 ± 0.0537 |
| XGBoost | 0.6871 ± 0.0083 | 0.6434 ± 0.0447 | 0.5771 ± 0.0176 | 0.5486 ± 0.0463 |
| Gradient Boosting | 0.6882 ± 0.0054 | 0.6407 ± 0.0428 | 0.5611 ± 0.0183 | 0.5464 ± 0.0437 |
| SVM | 0.5975 ± 0.0233 | 0.5633 ± 0.0271 | 0.5144 ± 0.0187 | 0.4925 ± 0.0426 |

Supplementary Table 9. Changes in exploratory outcomes during the treatment period.

| **Time** | **N, Cumulative adjusted mean**±**SD** | | | |  |
| --- | --- | --- | --- | --- | --- |
|  | **1 year** | **2 years** | **3 years** | **4 years** | **5 years or more** |
| **Change of CCT per year(um/year)** | 63, 1.79±5.66 | 45, 1.17±5.98 | **33, 0.90±4.52** | 30, 1.77±5.47 | 12, 0.93±8.32 |
| **Change of ACD per year(mm/year)** | 44, 0.00±0.20 | 44, 0.00±0.07 | 37, 0.02±0.09 | 30, -0.02±0.09 | 12, 0.04±0.06 |
| **Percentage of average annual change in ACD (%)** | 44, 0.46±6.51 | 44, 0.04±2.38 | 37, 0.65±2.79 | 30, -0.61±2.73 | 12, 1.13±1.75 |
| **Change of NCT per year, mmHg/year** | 609, 0.14±2.64 | 396, 0.24±2.42 | 173, -0.10±2.43 | 21, -0.35±3.01 | 1,  . |
| **Proportion of NCT within the normal range (%)** | 91.0% | 89.4% | 89.2% | 96.7% | 50.0% |
| **Change of LT per year(mm/year)** | 60,  -0.03±0.16 | 44,  0.00±0.08 | 37,  0.00±0.07 | 30,  0.02±0.10 | 12,  -0.02±0.08 |
| **Percentage of average annual change in LT (%)** | 60,  -0.77±4.07 | 44,  0.00±2.20 | 37,  0.02±2.10 | 30,  0.48±2.87 | 12,  -0.65±2.30 |

Supplementary Fig.1. Feature importance plots of the optimal model for Years 1–4. The height of each bar represents the relative contribution of the corresponding feature to the model’s predictive performance. (a) Year 1; (b) Year 2; (c) Year 3; (d) Year 4

Supplementary Fig.2. Alluvial diagrams for model prediction results and processes. Flow from features to predicted categories, constructed based on feature contributions to predictions calculated using SHAP values. Left nodes correspond to the N most influential features; right nodes correspond to all predicted categories. The width of connecting lines indicates the contribution proportion of each feature to its corresponding category. (a) Year 1; (b) Year 2; (c) Year 3; (d) Year 4
